# The Surgeon’s Relation to Homicides before the Courts

**Published:** 1886-11

**Authors:** 


F. E. DANIEL, M. D., EDITOR AND PUBLISHER.

COLLABORATORS:

*E. J. Doering, M. D., Chicago.*

*Geo. Cupples, M. D., San Antonio.*

*Odo Betz, M. D., Germany.*

*E. J. Beall, M. D., Fort Worth, Texas.*

*T. C. Osborn, M. D., Cleburne, Texas.*

*Wm. Penny, M. D., New York.*

*H. O. Marcy, M. D., Boston.*

*C. K. Gregg, M. D., Mexico.*

*R. M. Swearingen, M. D., Austin.*

*C. H. Wilkinson, M. D., Galveston.*

*J. W. McLaughlin, M. D., Austin.*

*R. H. L. Bibb, M. D., Mexico.*

### THE SURGEON'S RELATION TO HOMICIDES BEFORE THE COURTS.

In view of certain well known occurrences before the Courts here in Texas, and of a certain phase of our criminal law, it is small wonder that in "shooting scrapes"—as the not infrequent homicides are called, it is sometimes difficult to get a physician; physicians are gettiug to be, and with reason, very shy of going to a wounded man—especially at night, and more particularly to a colored man;—for they see before them the prospect of a frequent and long attendance upon first, the magistrates, then the District Court;—one sees himself on the witness stand under the galling fire of a cross examination, which will throw the bravest into a cold sweat; and he sees himself emerge, very much ruffled, without pay,—other, perhaps, than that of an ordinary witness to facts—(about laborer's wages)—when, may be, he has been made to "suppose a case"—and to give *an opinion*; and he may leave that stand a damaged, perhaps a ruined man, by having been angered or confused by the interrogations of a too zealous attorney, who will do much to discredit a witness, and clear, or convict—as the case may be—the prisoner at the bar.

But physicians do go, and do attend to the wounded, however reluctantly. They cannot disregard altogether the demands of humanity and of public opinion.

One of the well known occurrences alluded to is, briefly stated, about this: A man was struck and fell, striking heavily on some object. The surgeon found on the back of the head a swelling which crepitated on pressure. The patient was in a state of profound coma from, apparently, cerebral pressure. The only other evidences of injury about the person visible, was a drop of blood on the temple, which was overlooked. Believing the occipital bone to be fractured and depressed, the surgeon cut down on the "bump," and applied the trephine; nothing there. The man died. A knife blade was found in the brain, having been driven in in the temporal region and broken off, and an abscess had formed. The defendant was cleared on the ground that it was probable that the surgical operation, and the failure to remove the blade, were the cause of the death.

The peculiar phase of the law referred to is found in the judge's charge in the above case, (Texas Reports.) It seems that if it can be shown that the wounded man did not have done for him all that could have been done by the most skillful and competent surgeon—all that is known to the resources of the science and art of medicine, the defendant shall have the benefit of the omission or neglect; and if a grave operation has been performed, it shall be taken into consideration as a factor in the death. This seems just. To illustrate: A inflicts a slight cut on B on the fore-arm, severing the radial artery. A goes off and procures a physician who, instead of ligating the artery, makes some styptic application, and in course of time, the man bleeds to death. It is very clear that what is, in that case, technically a homicide, would have been, if the man had been properly treated, only an assault, and the physician is culpable. But granting that all has been done that is known to surgery, and the man still dies, the defense can be set up that the surgeon, and not the defendant killed him!

Before the District Court in this city at last term, the case of Dr. Felder, charged with killing Sam Pearson, colored, came up. (A report of the surgical part of the case was published in this JOURNAL in September, with cuts of the fractured vertebræ.—"Gunshot Fractures of the Spinal Column by W. J. Burt, M. D." A copy of the JOURNAL, containing it, was introduced into Court to illustrate the nature of the wound.)

It will be remembered that the ball fractured the body of one of the lumbar vertebræ producing paralysis of the lower extremities. An incision was made, but the ball was not found. After death—

which occurred some six or eight weeks later, the ball was found to be impinging on the cord, but the membrane was not ruptured. The man was attended by some of the best surgeons in Austin. The defense set up was, that the patient did not have proper surgical treatment, and it was claimed that the ball should have been removed; that had it been removed at once, the patient might have recovered. Two of the surgeons admitted the plea, (others thought it was a mortal wound and that it was of little consequence whether the ball were removed or not.) We are told that the bombarding of the first mentioned surgeon by the prosecuting attorney was terrific. "So you admit, Doctor," said he, "that the ball should have been removed?" "Yes." "Well sir, why did you not remove it?" "Because, had I done so, and the patient had died, I should have been considered accessory to his death" replied the Doctor. "So, then," said the attorney. "You were trying to *save yourself, and not the patient!*"

The Doctor was right. In order to remove the ball, trephining of the body of the vertebra would have been necessary, an operation of great gravity, usually attended with unfavorable results. It was not *known* before death that the cord was not severed; and had the surgeons trephined for the removal of the ball, and the patient had died,—which is more than likely,—the probabilities are largely in favor of the defendant's having been acquitted, and suspicion thrown on the susgeons. This looks hard. A man is shot in the abdomen. Shock and other evidences of wounded intestines convince the surgeon that only by an operation is there a chance for the safety of the patient. Yet, if he operates, and fails to save the patient, the defense is possible that the operation was a factor in the death; because it can be shown that quite as many,—many more, in fact,—gunshot wounds in the abdomen have recovered without operation than with it. Up to date, there are only three successful laparotomies for such wounds on record, to-wit: two by Dr. W. T. Bull, and one by Dr. J. B. Hamilton, (Dr. Bacon Saunder's case, of Bonham, has not been recorded); on the other hand, failure to operate may be pleaded successfully in defense of accused.

The above is given as a specimen of what a physician has to suffer on the witness stand in the surgery of homicides in Texas, for the $1.50 a day; and also to show the fearful alternative of doing his duty, and running the risk of a criminal prosecution himself, while aiding, unintentionally, to defeat justice, and clear the criminal, or shrinking from it, and being ridiculed in court!

We do not claim that this case is a parallel of the trephining case mentioned, nor that the trephining was necessary or justifiable; it was a mistake—to which the most careful are liable,—but the principle is the same, and serves to illustrate the predicament of the surgeon before the courts, and the operation of the law—sometimes to defeat justice. It seems the surgeon is liable, if he operates—and the prisoner has the benefit,—and equally so, if he fails to operate. He is thus made the scape-goat, and the accused often goes clear.

(In our last number Dr. Schmidt gives his experience before the courts as an expert in a rape case. In the same issue Dr. Peyton Turner, at Abilene, tells how he came near being indicted, because he performed craniotomy. At present, proceedings are being had, to indict two leading physicians of an adjoining county, for causing the death (?) of a woman, on whom the operation of laparotomy had been performed,—and for the death of a patient from chloroform, during preparation for an operation. In the former case, the woman got out of bed the day after the operation, and with regard to the latter—all surgeons know that no amount of skill and forethought will surely prevent such an occurrence, in even the healthiest persons, sometimes.)

These things all considered, is it a wonder that surgeons are afraid to do what their conscience, and their learning tell them is clearly their duty? And that they are shy of attending a wounded man?

Thus it comes about that through the operation of a well-meant law, instead of the skill and the intelligence of the physician being made an adjunct to the execution of justice—he is alienated, and in a kind of self-defense, he avoids the witness stand whenever it is possible to do so.

